# Experience of treating *Candida auris* cases at a general hospital in the state of Qatar

**DOI:** 10.1016/j.idcr.2020.e01007

**Published:** 2020-11-12

**Authors:** Adila Shaukat, Nasir Al Ansari, Walid Al Wali, Edin Karic, Ihab El Madhoun, Hassan Mitwally, Manal Hamed, Feah Alutra-Visan

**Affiliations:** a Infectious Disease Section, Department of Medicine, Al Wakra Hospital, Hamad Medical Corporation (HMC), Qatar; b Infection Prevention and Control Department, Al Wakra Hospital, HMC, Qatar; c Department of Laboratory Medicine and Pathology, HMC, Qatar; d Critical Care Dept, Al Wakra Hospital, HMC, Qatar; e Medicine Division, Al Wakra Hospital-HMC, Qatar; f Pharmacy Department, Al Wakra Hospital, HMC, Qatar; g Department of Microbiology, HMC, Qatar

**Keywords:** Candida auris, Candidemia, Urinary tract infection, Respiratory tract infection, Colonization, Skin infection.

## Abstract

**Background and objectives:**

So far there have been no studies on *Candida auris* in Qatar. This study aimed to describe the clinical spectrum and outcome of *C. auris* infection in patients admitted to a general hospital in Qatar.

**Methods:**

We conducted this descriptive observational study in a general hospital in Qatar. We have involved all patients with *C. auris* infection and colonization admitted to a general hospital from December 2018 to August 2019.

**Results:**

We identified 13 patients with confirmed *C.auris* infection/colonization, of which five cases represented an actual *C. auris* infection, while the remaining eight cases were considered as colonization. The mean age of the patients with infection was 76.6 ± 8.4 years, while the mean age of the patients with colonization was 66.4 ± 24.7 years. Among the individuals clinically infected with *C. auris*, two had urinary tract infections, one had candidemia, one acquired soft tissue infection, and one had a lower respiratory tract infection. All strains of *C. auris* were susceptible to echinocandins, flucytosine, and posaconazole while resistance to fluconazole and amphotericin B. Of the patients with *C. auris* infection who received systemic antifungal therapy, three (60%) died during antifungal therapy.

**Conclusion:**

Our study showed that *C. auris* can cause a wide variety of invasive infections, including bloodstream infection, urinary tract infection, skin infection, and lower respiratory tract infections, especially in critically ill patients. In addition, our isolates showed resistance to the most common antifungal agents such as fluconazole and amphotericin B.

## Highlights

• *Candida. auris* can cause invasive infections, including bloodstream, urinary tract, skin and ssoft tissue and lower respiratory tract infections.
• Identification of *C. auris* requires specialized laboratory methods.
• *C. auris* is associated with high morbidity and mortality.
• *C. auris* isolates were resistant to the common antifungal agents such as fluconazole and amphotericin B.

## Introduction

*Candida auris* is a novel multidrug-resistant yeast with high overall mortality that was first isolated from the external auditory canal of a patient in Japan in 2009 [1]. Since then, this fungal infection has been reported from various countries across the world [[2], [3], [4]], and over time it has become a serious global health concern as one of the most serious emerging pathogens that critical care physicians should be aware of [5].

*C. auris* being resistant to major antifungal classes used to treat Candida including azole antifungal agents, poses a challenge to routine microbiology laboratories, as *C. auris* can be misidentified with standard laboratory techniques, and have a tendency to cause outbreaks in healthcare settings especially critical care areas despite adequate infection prevention and control measures [4,5].

In Qatar, there is no published data on *Candida aurisso far*. In this series, we reported the first outbreak of *C. auris* infection in Qatar, to describe the clinical spectrum and outcome of this infection in the affected patients.

## Methods and patients

We conducted this descriptive observational study in a general hospital in Qatar. We involved all patients with *Candida Auris* infection and colonization in the intensive care units and other wards from December 2018 to August 2019. This study was given ethical approval by the medical research committee at Hamad Medical Corporation, under number: MRC-01-19-503..

## Definitions

Colonization is defined as isolation of *C. auris* from endotracheal aspiration fluid, throat swabs, sputum, urine, and samples from central venous catheters or other parts of the body in absence of clinical signs or symptomes of infection. *C.auris* infection is defined as the isolation of C*auris* from clinical specimens with compatible clinical signs and symptoms of infection [5].

## *Candida auris* identification

All clinical specimens, from different sites, were cultured by quantitative technique on Sabouraud Dextrose Agar (OXOID, UK) and incubated at 35−37 °C for 48 h. Preliminary fungal strain identification was based on colony morphology on Chromogenic Candida Agar (OXOID, UK), while the identification to the species level was confirmed by Vitek 2 XL automated system (bioMerieux). Susceptibility of strains to Amphotericin B, Fluconazole, 5-fluorocytosine, and voriconazole was determined by using Sensititre™ YeastOne™ plate and by interpreting results according to closely related Candida species and on expert opinion. As per the Centers for Disease Control and Prevention (CDC), there are currently no established *C. auris*-specific susceptibility breakpoints [6].

Pulsed-field gel electrophoresis (PFGE) typing, which consisted of electrophoretic karyotyping (EK), was performed to compare the isolates from different sites. Following the results of the PFGE, an outbreak of *C. auris* infection in critical care unit and medical unit was confirmed by identifying five5 cases and patient screening revealed colonization of eight additional patients. Intensive efforts were done to find out the cause of cross-transmission and environmental and surface swabbing was done in affected areas, but all results were negative.

## Data analysis

The results of analyses of continuous variables are expressed as means and standard deviations (SD) unless otherwise specified.

## Results

During the study period, we identified 13 patients with confirmed *C. auris* infection/colonization, of which five cases represented an actual Candida infection, while the remaining eight cases were considered colonization. The mean age of the patients with infection was 76.6 ± 8.4 years (range: 65–90 years), while the mean age of the patients with colonization was 66.4 ± 24.7 years (range: 23–91 years). Table 1 describes the demographic characteristics of the patients involved in this study.

**Table 1:** Candida auris infection/colonization patients details.

Case/No	Age	Sex	Site of infection/ or site of Candida isolation	Type of infection	Pre or co-infection	Co-morbidity	Treatment provided	Outcome
1	78 years	Male	Tracheal aspirate and urine	Lower respiratory tract infection	Corona virus 229 E PCR positive from nasal swab	Interstitial lung disease	Anidulafungin	Died of hypoxic respiratory failure
2	79 years	Male	Nose and decubitus ulcer	Skin soft tissue infection	Pseudomonas MDR and Morganella morganii from decubitus ulcer	Diabetes mellitus, sacral bed sores	Flucytosine	Died of bacterial/fungal sepsis
3	71 years	Male	Nose, throat, tracheal aspirate, and decubitus ulcer	Candidemia	*Pseudomonas aeruginosa* MDR and ESBL *Klebsiella pneumoniae* from sputum	Diabetes mellitus, sacral bed sores	Anidulafungin and posaconazole	Cured
4	90 years	Male	urine, throat and nose	Urinary tract infection	*Klebsiella pneumoniae* and carbapenem resistant *Pseudomonas aeruginosa* from sputum, *Pseudomonas aeruginosa* MDR from a bedsore	Cerebrovascular accident, dementia	Anidulafungin	Cured
5	65 years	Male	Throat, sputum, groin and urine	Urinary tract infection	*Pseudomonas aeruginosa multidrug resistant*	Motor neuron disease, hospital-acquired pneumonia	Anidulafungin	Died of bacterial pneumonia
6	29 years	Male	Groin	Colonization	ESBL Klebsiella	Acute liver failure secondary to hepatitis C, acute kidney injury, critical care polyneuropathy	Terbinafine spray	Discharged home
7	86 years	Male	Axilla, urine	Colonization	Pseudomonas aeruginosa	COPD, vascular dementia, bedbound on tracheostomy to	Terbinafine spray, nystatin application	Died due to aspiration pneumonia and hypoxic respiratory failure
8	80 years	Female	Nose, tracheostomy site	Colonization	ESBL Klebsiella	Chronic kidney disease, coronary artery disease, on tracheostomy	Terbinafine spray, nystatin application	Transfer to geriatric ward
9	62 years	Female	Axilla	Colonization	Pseudomonas multi drug-resistant	Chronic kidney disease, necrotizing fasciitis	Terbinafine spray, nystatin application	Died due to bacterial sepsis
10	91 years	Female	Groin area	Colonization	None	COPD, hypertension	Terbinafine spray, nystatin application	Discharged home
11	23 years	Male	Nose, axilla	Colonization	Escherichia coli	Hypoxic brain injury, recurrent urinary tract infection	Terbinafine spray, nystatin application	Transfer to long-term unit
12	75 years	Male	Nose, groin	Colonization	Pseudomonas aeruginosa	Diabetes mellitus, chronic kidney disease, recurrent pneumonia	Terbinafine spray, nystatin application	Discharged home
13	85 years	Male	urine	Colonization	Pseudomonas aeruginosa	Parkinson’s disease, cerebrovascular accident	Terbinafine spray, nystatin application	Transfer to geriatric unit

PCR: polymerase chain reaction, MRD: multi-drug resiatant, ESBL: extended spectrum beta lactamase, COPD: chronic obstructive pulmonary disease.

Among the individuals clinically infected with *C. auris*, two had urinary tract infections, one had candidemia, one acquired soft tissue infection, and one had a lower respiratory tract infection. All patients had bacterial or viral infections prior to or concomitantly with *C. auris* infection/colonization, as shown in Table 1.

**Fig. 1:**
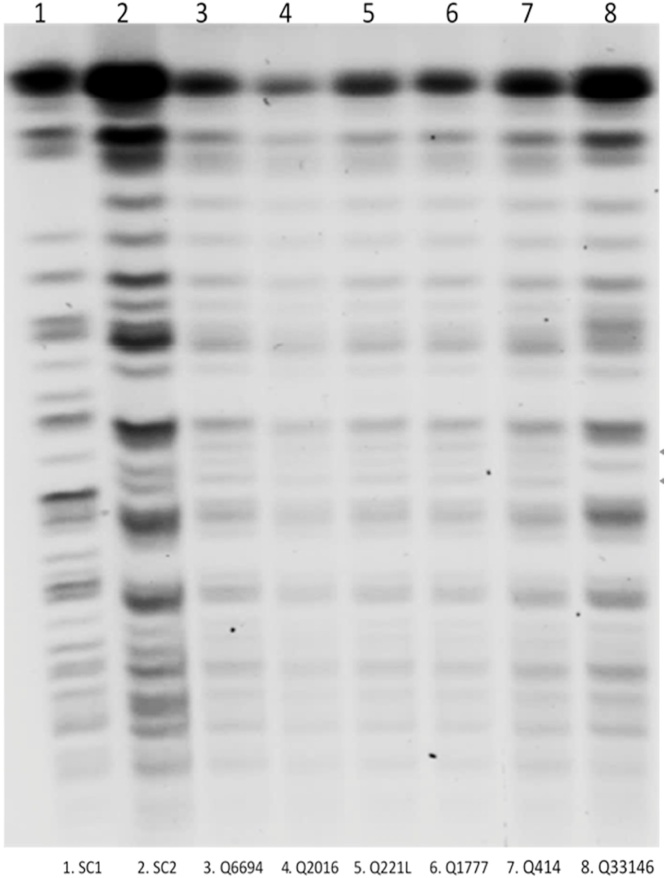
Electrophoretic karyotypes of *C. auris* isolates. Karyotypes of representative outbreak isolates from five patients in the intensive care unit. Lane 1, 2 and 8 are control specimens which served as comparison for different genotypes. Lane 3 to 7 strains (specimens from the five *C. auris* cases) show no single band variation and are likely representing the same strain.

For the typing of *C. auris* isolates, the molecular technique PFGE, which consisted of electrophoretic karyotyping (EK), was utilized to compare the isolates from different sites. The PFGE karyotype of the outbreak isolates of *C. auris* in our series is shown in Fig. 1. Antifungal susceptibility tests were performed on isolates from infected subjects. All strains of *C. auris* shared the same susceptibility profile, being susceptible to echinocandins (especially anidulafungin), flucytosine, and posaconazole while resistance to fluconazole and amphotericin B. Table 2 shows the susceptibility pattern in the form of minimal inhibitory concentrations (MIC) of antifungal agents for the *C. auris* isolates. All patients with *C. auris* infection received systemic antifungal drugs, while the eight patients who were colonized were appropriately decolonized with topical nystatin and terbinafine as recommended by the CDC (Table 1).

**Table 2:** Susceptibility pattern in the form of minimal inhibitory concentrations (MIC) of antifungal agents for the *C. auris* isolates from subjects with infection.

Antifungal drugs	Patient 1	Patient 2	Patient 3	Patient 4	Patient 5
Amphotericin	4-R	4-R	2-R	4-R	2-R
Caspofungin	0.25	8-R	8	0.5	8
Fluconazole	64	128-R	128-R	128-R	128-R
Flucytocin	0.125	0.5-S	0.12	0.12	0.12
Itraconazole	0.125-R	16-R	0.12	0.12	16
Posaconazole	0.012	8-R	0.06	0.06-S	8
Voriconazole	0.25	8-R	0.25	0.5	8
Anidulafungin	0.125-S	0.5-I	0.25	0.12-S	0.5-I
Micafungin			0.25	0.12	0.25

R: resistant, S: sensitive, I: intermediate.

Among the patients with *C. auris* infection who received systemic antifungal therapy, three (60 %) died during antifungal therapy. The other two patients were successfully treated and appropriately decolonized of *C. auris* (Table 1).

## Discussion

Recent reports showed that *C. auris* is an emerging yeast that has been identified worldwide as a cause of severe invasive healthcare infections, which mostly affect critically ill patients and cause substantial morbidity and mortality [7,8]. To our knowledge, our series is the first designed to study this infection in Qatar.

Many *C. auris* outbreaks have been reported worldwide. In India, the first *C. auris* outbreak was reported in 2013 by Chowdhary et al. [9] who identified 12 patients with positive microbiological clinical specimens collected between 2009 and 2012. While Calvo et al. reported the first outbreak of *C. auris* infection in Venezuela between March 2012 and July 2013 [10]. All the isolates were initially identified as *C. haemulonii*. However, the isolation of *C. auris* was later confirmed by genome sequencing [9,10]. Similarly, we have reported the first outbreak of *C. auris* infection in Qatar, identifying 13 patients. The emergence of *C. auris* in our hospital raises concerns that this fungus may spread to other healthcare settings, particularly critical care facilities in Qatar, requiring intensified measures to control the spread of this infection. Therefore, knowing the source of infection and detection of possible routes of transmission can help in preventing the clonal spread of this infection and hospital outbreaks among various health facilities in Qatar [5,7,8]. Similarly, intensive efforts have been made in our hospital to find the cause of the cross-transmission. Environmental and surface swabs were carried out in the affected areas, but all results were negative.

Diagnosing *C. auris* infection is difficult because the clinical presentation is non-specific or may not be recognizable since patients infected with *C. auris* often have another serious illness or condition. Moreover, *C. auris* can be misidentified with standard laboratory techniques as *C. haemulonii* [11,12]. As a result, a high index of suspicion is required to diagnose this infection. In addition, accurate identification of *C. auris* through specialized laboratory methods is required to avoid misidentification and inappropriate treatment that may make it difficult to control the spread of *C. auris* in the healthcare settings [10]. In this study, the diagnosis of C. auris infection was suspected because of the resistance of the isolates to fluconazole and amphotericin B. The diagnosis was confirmed by molecular methods.

The spectrum of *C. auris* infection ranges from superficial infections that affect the skin to widespread infections that affect the brain, heart, lungs, liver, spleen, and kidneys [5]. Antifungal therapy should be administered to eradicate and control *C. auris* infection. On the other hand, *C. auris* can be isolated from the skin, rectum, wounds or mouth of some patients who do not show symptoms of infection. This condition is referred to as asymptomatic colonization and treatment with antifungal drugs does not eradicate *C. auris* colonization. However, the identification of *C. auris* colonization is significant because it carries the risk of transmission, which requires the immediate implementation of adequate infection control measures [13]. Likewise, our patients showed different clinical presentations, and cases with colonization were identified and appropriately decolonized with topical nystatin and terbinafine as recommended by the CDC.

In agreement with other reports [[3], [4], [5],^7^,^13^], our isolates showed resistance to the most important antifungal agents such as fluconazole and amphotericin B. The all cause mortality among our patients was 60 % which is in line with the mortality rate seen in other studies ranging from 30 to 60% [3].

One of the limitations of this study is the retrospective nature of the research. In addition, the small sample size is another factor that limits the generalizability of these findings.

## Conclusion

*C. auris* can cause a wide variety of invasive infections, including bloodstream infections, urinary tract infection, skin infection, and lower respiratory tract infection, especially in critically ill patients. In addition, all isolates showed resistance to fluconazole and amphotericin B and were sensitive to echinocandins especially anidulafungin.

## Authors contribution (Authorship)

**Adila Shaukat**: Desgning, interpretation of data, revising and approving the final draft.

**Nasir Al Ansari**: conception of the study, revising and approving the final draft.

**Walid Al Wali**: interpretation of data, revising and approving the final draft.

**Edin Karic**: interpretation of data, revising and approving the final draft.

**Ihab El Madhoun**: acquisition of data, revising and approving the final draft.

**Hassan Mitwally**: interpretation of data, revising and approving the final draft.

**Manal Hamed**: acquisition of data, revising and approving the final draft.

**Feah Alutra- Visan**: interpretation of data, drafting the article and approving the final draft.

## Conflict of interest

All authors report no conflict of interest.
